# Infodemics: Do healthcare professionals detect corona-related false news stories better than students?

**DOI:** 10.1371/journal.pone.0247517

**Published:** 2021-03-10

**Authors:** Sven Grüner, Felix Krüger

**Affiliations:** 1 Institute of Agricultural and Nutritional Sciences, Martin Luther University Halle-Wittenberg, Halle (Saale), Germany; 2 Faculty of Law and Economics, Martin Luther University Halle-Wittenberg, Halle (Saale), Germany; Universitat Luzern, SWITZERLAND

## Abstract

False news stories cause welfare losses and fatal health consequences. To limit its dissemination, it is essential to know what determines the ability to distinguish between true and false news stories. In our experimental study, we present subjects corona-related stories taken from the media from various categories (e.g. social isolation, economic consequences, direct health consequences, and strong exaggeration). The subject’s task is to evaluate the stories as true or false. Besides students with and without healthcare background, we recruit healthcare professionals to increase the external validity of our study. Our main findings are: (i) Healthcare professionals perform similar to students in correctly distinguishing between true and false news stories. (ii) The propensity to engage in analytical thinking and actively open-minded thinking is positively associated with the ability to distinguish between true and false. (iii) We find that the residence of the subjects (East- or West-Germany) plays only a minor role. (iv) If news stories are in line with existing narratives, subjects tend to think that the stories are true.

## 1 Introduction

The corona crisis has provided many examples of what Tedros Adhanom Ghebreyesus, Director-General of the World Health Organization [1], denoted as an “infodemic.” For example, it has been suggested to cure Covid-19 with the help of smoking, cocaine, or even cow urine. Hundreds of Iranians died from drinking methanol to cure Covid-19 and many others suffered from serious health implications [2]. There are also adventurous explanations for its roots, including bioweapon or the 5G wireless technology [3]. Moreover, conspiracy theories and false claims went viral when, for example, about 20,000 people were demonstrating in Berlin (Germany) in June 2020 against the corona measures of chancellor Merkel. In social networks, some people (mostly supporters of right-wing parties) shared postings that a lot more people would have joined the demonstration. These unedited news stories are dangerous in that they give the impression that a lot more people are against these measures. As a consequence of the rise of corona-related false news stories, various state governments and institutions have taken action. For example, the German Federal Ministry of Health released a warning against covid-19-related false news stories [4]. Twitter removed a tweet of President Donald Trump in which he retweeted a way to supposedly cure from Covid-19, when in fact no such way existed [5]. In several countries, laws against the spread of false news have been passed. In Hungary, for example, people can be sentenced to prison for up to 5 years if they violate the law, which, however, creates an atmosphere of uncertainty among journalists [6].

What’s the problem with false news stories in general? Lazer et al. [7] argue “We define “fake news” to be fabricated information that mimics news media content in form but not in organizational process or intent. Fake-news outlets, in turn, lack the news media’s editorial norms and processes for ensuring the accuracy and credibility of information.” We are in line with this definition but prefer to speak of *false news stories* throughout this paper. The reason is that the term fake news has not only been used to describe false information but also derogatory for information that does not reflect one’s own (e.g. of a politician) opinion [8]. Besides terminology, a key problem of false news stories is that they restrict the functioning of markets as well as democratic, political decision processes. They prevent competition of ideas what, in turn, can lead to societal misallocations, for example, by influencing public opinion and voting [9]. Moreover, trust in media and institutions in general can be eroded. Even worse, false news stories are spreading fast. For example, Vosoughi et al. [10] found in their Twitter study that false news spread faster than true news since the former are mostly topical and cause emotional reactions. That makes it hard to correct them, in particular when a huge amount of false news information is generated as it is the case in the corona pandemic [11].

Since its outbreak, there has been a huge amount of information on Covid-19 every day [12]. Many news items were correct, but there were also a large number of false news stories. Thus, there is no surprise that Donovan [13] calls in a Nature article that “Social-media companies must flatten the curve of misinformation.” To avoid welfare losses in general and adverse health consequences due to false claims in particular, it is socially desirable that people are able to distinguish between true and false news information. But who is good at this exercise? The objective of this paper is to identify determinants that help to distinguish between true and false news stories. Knowledge about such determinants can help to reduce the dissemination of false news stories.

This question is not new. For example, Pennycook and Rand [14,15] have tackled it before. However, our study differs from former studies in the design and context of the news stories. While other studies mostly present headlines of a news story, we show experimental subjects also a couple of sentences or a short paragraph. Instead of analyzing political news stories (e.g. Presidential Election Campaign; [14,15]), we address corona-related news stories. Furthermore, many experimental studies deal with students only [cf., 16–18]. Students are easy to recruit because of their low opportunity costs. However, the external validity of a study can be questioned if only students are considered since they are rather an untypical population with regard to age, income, and education. We do not restrict ourselves with the population of students but also recruit healthcare professionals to increase the external validity of our study. It seems to be an interesting question to ask whether the expertise and experience of healthcare professionals help to more adequately process corona-related news information.

The rest of the paper is structured as follows. In Section 2, we describe our behavioral research questions. After presenting the methods (experimental design, approach to data analysis, recruitment procedure) in Section 3, we describe our results in Section 4. Section 5 concludes.

### 2 Behavioral research question

As indicated above, this paper aims at analyzing the ability to distinguish between true and false corona-related news stories. We tackle the following research questions:

*i. Are students who are enrolled in medicine or healthcare perform better in identifying false news stories than other students*? *Do healthcare professionals (e*.*g*. *physicians) perform best in differentiating between true and false news stories*?

According to Pennycook et al. [19], COVID-19 is a scientific issue. We expect that students of medicine and the health care sciences are more capable of processing and classifying information on corona due to their theoretical knowledge than students of other degree programs. Thus, the former should be better in distinguishing between true and false news stories. We assume that healthcare professionals (e.g. physicians) are best at differentiating between true and false news stories by virtue of their theoretical knowledge and practical experience.

*ii. Are the propensity to engage in analytical reasoning (= cognitive sophistication) and actively open-minded thinking (AOT) positively correlated with the ability to correctly distinguish between true and false news*?

Pennycook and Rand [14,15] find that the propensity to engage in analytical reasoning helps to differentiate between true and false news stories. In line with that, analyzing data from Canada, the U.K., and the U.S.A., Pennycook et al. [19] find a negative association between cognitive sophistication and misperceptions about COVID-19. Another safeguard against false news stories is actively open-minded thinking: experimental evidence has shown that there is a positive correlation between the AOT score and the ability to differentiate between true and false news stories [20].

*iii. Does the familiarity with the stories (i*.*e*., *subjects report that they have seen the story before) increase the probability that people think that the story is true*?

In their experimental study, Pennycook et al. [21] show that even a single exposure increases the perceived accuracy of false news stories. The repetition of a news story promotes familiarity and higher familiarity increases, in turn, the probability that it is perceived as true [22]. This is also known as the illusory truth effect. Moreover, experimental results in the field of environmental economics provide evidence that even the perception of having seen a news story before, increases the likelihood that the story is considered to be true [23]. As a consequence, false news stories are more likely to be accepted as true. We investigate this relationship in the context of COVID-19.

*iv. Are there differences between the eastern and western population of Germany in the perception of news stories*?

Before reunification, Germany experienced two distinct economic systems: capitalism in West Germany and socialism in East Germany. The different socialization could lead individuals to perceive information differently and reacting in a different manner on political measures (e.g. lockdown). Different socialization and experiences of corona may have led to different emotions and evaluations of media content. However, it remains unclear whether this affects the ability to differentiate between true and false news.

*v. How do anxiety and personal experiences influence the ability to distinguish between true and false news stories*?

Confirmation bias assumes that individuals are more likely to believe information that is consistent with their own views [e.g. 24]. Similarly, people who are afraid of corona could be more likely to believe news stories that stress the negative consequences of corona. Similarly, personal experiences and involvement may be relevant: the more affected an individual is, the more likely he or she is to uncritically accept news items that address strong negative consequences. However, it remains an open question whether fears or personal experiences are important determinants to explain the ability to distinguish between true and false news stories.

## 3 Methods

This study has been approved by the German Association for Experimental Economic Research e.V. (No. 8ScdfpyT). The participants were informed about the background of the study (problem of false news stories in the health-care sector) and what had to be done in the study (to evaluate news stories, answer questions about experiences and opinions, etc.). They were also told that participation is voluntary and that data processing is anonymous and confidential. The study is in line with the General Data Protection Regulation (EU) 2016/679. To participate, individuals had to confirm (by actively checking the respective boxes in the web-based study) that they are at least 18 years old and accept the conditions of participation. Moreover, the study has been pre-registered before any data have been collected (AsPredicted #40327).

### 3.1 Experimental design

The study consists of two parts: In the first part, experimental subjects are shown 8 stories taken from the news media. Note that the study was launched in May 2020 and the news stories, therefore, reflect the early stages of the corona pandemic. In the second part, we collect data on a variety of socio-demographic variables, attitudes, and personality traits.

#### A. News stories

We present each experimental subject with 8 corona-related stories taken from the media (cf., Table 1 for a short description of the stories; the sources of the stories can found in Table A1 of the Appendix). They were presented a headline and a couple of sentences (e.g. a small paragraph) of a news article. By not only showing a headline but also a couple of sentences, we provide the subjects with background information. For example, Germans who are not living in Saxony-Anhalt may never have heard about Haseloff. Thus, subjects can read some details about a topic if they want. In reality, people can also look for additional information, for example, by using a search engine. However, we cannot say if there are differences at all between our design and only providing headlines. This is left open for further research.

**Table 1:** News stories of the experiment (short version).

#	Label	Short description of the *true* story	Short description of the *false* story
1	*East Germany and Corona*	*Haseloff*: *East Germans are better prepared for the corona crisis than West Germans*.	*Haseloff*: *East Germans are* ***less well*** *prepared for the corona crisis than West Germans*.
		According to Saxony-Anhalt’s Prime Minister Reiner Haseloff (CDU), East Germans can withstand hard periods of time. State authority is more accepted on the territory of the former GDR.	According to Saxony-Anhalt’s Prime Minister Reiner Haseloff (CDU), East Germans **don’t let them locked up as they were in the GDR**. State authority is **less** accepted on the territory of the former GDR.
2	*Psychiatrists on social isolation*	*Social isolation*: *psychiatrists are warning*	*Social isolation*: *psychiatrists are* ***optimistic***
		The German Association for Psychiatry, Psychotherapy and Psychosomatics (DGPPN) warns of a rise in suicide rates if contact is blocked for a longer period. “Social isolation is a major stress factor and can exacerbate psychological disorders,” said Andreas Heinz, the president of the DGPPN.	The German Association for Psychiatry, Psychotherapy and Psychosomatics (DGPPN) **is optimistic, even if contact is cut off for a longer period of time**. “Social isolation is a **stress factor, but it can also strengthen inner-domestic relationships and help us to recall fundamental values**,” said the president of the DGPPN, Andreas Heinz.
3	*Germany’s medical care dependency on Asia*	*Germany’s medical care is not at all dependent on Asia*	*Germany’s medical care* ***heavily dependent*** *on Asia*
		“The thesis that Germany is on the drip of globalization in the trade of medical goods cannot be empirically proven,” write the scientists Martin Braml, Feodora Teti and Rahel Aichele in an essay for the Ifo Schnelldienst.	“The thesis that Germany is on the drip of globalization in the trade of medical goods **is empirically verifiable**,” write the scientists Martin Braml, Feodora Teti and Rahel Aichele in an essay for the Ifo Schnelldienst.
4	*Beds in clinics*	*Clinics in the corona crisis*: *short-time work*	*Clinics in the corona crisis*: ***overtime work***
		Many beds are currently empty in numerous clinics in Germany. This is because plannable operations are postponed or canceled. As a result, the Schön-Klinik, for example, sent employees at several locations on short-time working, including physicians and nursing staff.	In numerous clinics in Germany, beds are **overcrowded even though scheduled operations are postponed or canceled**. As a result, the Schön-Klinik, for example, **obliges employees at several locations to work overtime**, including physicians and nursing staff.
5	*Gender and Corona*	*Men more vulnerable to corona due to an enzyme*	***Women*** *more vulnerable to corona due to an enzyme*
		Men are more vulnerable to the new coronavirus and die from it more often. The reason: The blood of men has a significantly higher value of the key enzyme ACE2 than the blood of women.	**Women** are more vulnerable to the new coronavirus and die from it more often. The reason: The blood of **women** has a significantly higher value of the key enzyme ACE2 than the blood of **men**.
6	*Efficacy of homeopathy*	*No protection through globules*	***Protection*** *through globules*
		Based on the available scientific studies, we can currently assume that homeopathic remedies themselves have no effect. In any case, they are nothing more than a sham drug that is completely free of active ingredients.	Based on the available scientific studies, we can currently assume that **homeopathic remedies themselves have a relatively high effect without simultaneous undesirable side effects**.
7	*Corona App*	*Track infected*, *preserve privacy*	*Track infected*, *preserve privacy*
		How could a Corona App fulfill its purpose and at the same time guarantee data protection? With the app technology behind the “Pan-European Privacy-Preserving Proximity Tracing” initiative (PEPP-PT), data is sent to a central server, which is operated by the state, for example.	How could a Corona App fulfill its purpose and at the same time guarantee data protection? With the app technology behind the “Pan-European Privacy-Preserving Proximity Tracing” initiative (PEPP-PT), data is sent to a central server, which is operated by the state or a **private provider such as Facebook, Google or Huawei**, for example. **In Germany, for example, there have already been negotiations with Facebook and Huawei.**
8	*Corona transmission by farts*	*Australian doctor points to farts when questioned about corona transmission*	*Australian doctor points to farts when questioned about corona transmission*
		The coronavirus can be transmitted by the droplets released when coughing—but also by a fart. At least that’s what Norman Swan, an Australian doctor and podcaster, says in a new episode of “Coronacast,” a coronavirus podcast of the Australian channel ABC.	The coronavirus can be transmitted by the droplets released when coughing—but also by a fart. At least that’s what Norman Swan, an Australian doctor and podcaster, says in a new episode of “Coronacast,” a coronavirus podcast of the Australian channel ABC. **Texas vice-governor Dan Patrick promptly announced a decree of a general pants duty. The stipulated minimum leg length should be 20 inches (approx. 51 cm). By wearing pants and underpants, the effect is similar to that of an everyday mask. The population in Germany is also reacting to this threat. On Twitter, there are not only contributions under #Mask duty but also under #Pants duty.**

The overall topics of the stories can be roughly divided into 4 categories: *Social isolation* (stories 1 & 2), *economic consequences* (stories 3 & 4), *direct health consequences* (stories 5 & 6), and *strong exaggeration* (stories 7 & 8). For each story, we have a true and a false version. We refer to a story as true if we did adopt the story from the media without manipulating its content. The false news stories contain any kind of false news information. After presenting the subjects a story (either true or false), we asked them whether they believe that the story is accurate (i.e., does not contain any kind of false news information). We randomly assign subjects either to the correct or false version of a story. Randomization allows us to interpret the results in terms of causality and not only correlation. Moreover, we attached three further questions to each story: (i) how confident are subjects in their assessment, (ii) have the subjects seen the news story before, and (iii) has the news story surprised the subjects when they read it. Overall, we randomized the order of the news stories to mitigate possible order and anchoring effects. For example, the news stories could cause emotions to an unknown extent, which might affect the response behavior to other stories.

What do the manipulations of the stories (i.e., false news stories) look like? With the exception of the category strong exaggeration, we have changed the sign of the core statement of each story. For example, the correct version of story 1 is about Saxony-Anhalt’s Prime Minister Reiner Haseloff who has argued that East Germans are better prepared for the corona crisis. The key message of the false story is reversed in the sense that the politician said that East Germans are less well prepared for the corona crisis. Let us take a look at another example, for illustration purposes. The fifth story, which deals with direct health consequences due to corona, is about gender. The correct version argues that men are more vulnerable to the new coronavirus. The false version, where we completely change the central message, claims that women are more vulnerable to the new coronavirus. The stories that we label as strong exaggeration are made much more extreme in the false version. For example, story 8 is about the transmission of corona via farts. In its correct version, it is only said that an Australian medical doctor has made some statements on this. In the false version, it is claimed that the Texas vice-governor initiated a general pants duty and that there is some Twitter activity (e.g. #Pants duty).

#### B. Further variables

*Propensity to engage in analytical reasoning*. Frederick [25] introduced a cognitive reflection test (CRT) to measure whether people can be described as intuitive or reflective thinkers. The test consists of a bunch of questions that have an intuitive but wrong answer. The correct answer can be found out at a second look (i.e., after rechecking the result). This test is often used to elicit the propensity to engage in analytical reasoning: a high score in this test is associated with analytical thinking, whereas a low score is related to intuitive thinking. We slightly changed the wording of the original items of Frederick’s test. For example, we asked: “A safety mask and a disinfectant product cost together €11.10. The protective mask costs €10 more than the disinfectant. How much does the disinfectant cost?” The correct answer reads €0.55 (an intuitive but false answer amounts to €1.10). *Actively open-minded thinking (AOT)*. AOT measures whether actively open-minded thinking is perceived as good. We adopt the 7-item scale from Haran et al. [26]. For example, one of their items is: “People should take into consideration evidence that goes against their beliefs.” Beyond CRT and AOT, we collected socio-demographic variables (e.g. age, education, gender, residence) and variables about fears and anxiety. The latter does not only include worries about immediate adverse health consequences, social isolation, and economic consequences but also individual actions as a consequence of corona (basic food reserves, hygiene products, and homeopathy). We also captured data on consumption of information and related attitudes (e.g. trust in media, change of trust in media, trust in government).

#### C. Financial incentives

We raffled 5 x €50 among all participants. In order to separate the answers given in the study and personal data, the participants were asked to send us an informal e-mail if they would like to participate in the raffle.

### 3.2 Statistical methods used for data analysis

Our variable of interest is whether subjects correctly identify news stories taken from the media. Correct identification means that correct news stories are identified as correct and false news stories are identified as wrong.

#### I. Overall correct identification

On the aggregate level, we sum up how often the stories are correctly identified by the subjects. Since we examine a total of 8 news stories, the dependent variable can take values from 0 to 8. This allows us to run a simple OLS regression. The regression contains *Population* (i.e., whether subjects identified themselves as healthcare professionals, healthcare students, non-healthcare students or something else). The category (i.e., vector) *Thinking* captures both CRT and AOT. Familiarity measures whether subjects have seen the stories before (i.e., it is aggregated over all stories), whether the stories are surprising and whether subjects are certain in their answering behaviors. The dummy *East* covers the residence of the subjects (East Germany or West Germany). *Anxiety&Involvement* is about the worries of the subjects (e.g. social isolation, immediate health consequences, consequences for the economy; reactions: food reserves or more disinfection; risk factors, such as smoking behavior, age). The other variables in the regression are for exploratory purposes/serve as controls. *Education* measures the highest formal degree of the subjects. *Gender* captures the gender the subjects identify with. *Information* measures the activities and perception of news and how they are communicated (e.g. trust in media, media consumption). *Week* controls for the point in time when the subjects joined the study and *Time* measures the number of minutes the subjects needed to finish the study.

$$Y(\sum Correct\;identification)=Population∙\beta_{1}+Thinking∙\beta_{2}+\sum Familiarity∙\beta_{3}+East∙\beta_{4}+Anxiety\&Personal\;experiences∙\beta_{5}+Education∙\beta_{6}+Gender∙\beta_{7}+Information∙\beta_{8}+Week∙\beta_{9}+Time∙\beta_{10}$$ 1

#### II. Story-by-story correct identification

On the story-by-story analysis, we look at each story separately (i.e., no aggregation over the stories). Since the dependent variable is binary we run logit regressions. To meaningfully interpret the results, we provide (average) marginal effects [27,28].

$$Y(Correct\;identification\;1/0)=Population∙\beta_{1}+Thinking∙\beta_{2}+Familiarity∙\beta_{3}+East∙\beta_{4}+Anxiety\&Personal\;experiences∙\beta_{5}+Education∙\beta_{6}+Gender∙\beta_{7}+Information∙\beta_{8}+Week∙\beta_{9}+Time∙\beta_{10}$$ 2

### 3.3 Recruitment strategy

We planned to recruit healthcare professionals, healthcare students, and non-healthcare students. The starting point to recruit students was a list of universities in Germany from Wikipedia (https://de.wikipedia.org/wiki/Liste_der_Hochschulen_in_Deutschland). From this list, we selected the largest universities (in terms of the number of students) and contacted the deans/deans of studies with the request to advertise the study. In addition, we directly contacted several professors from different departments and student councils. We put emphasis on covering subjects from different regions in Germany to obtain meaningful results (e.g. not only subjects from the south of Germany). In order to recruit healthcare professionals, we used the publicly available physician lists of the Association of Statutory Health Insurance Physicians of various federal states of Germany. Furthermore, subjects from various university hospitals were considered as long as contact details are publicly available.

### 3.4 Data manipulation

Before analyzing the data, we carried out some plausibility checks. As a result, we dropped a total of three subjects. Two of them were fast straightliners who took less than 5 minutes to finish the study. The third subject gave implausible answers (e.g. age = 99).

## 4 Results

### 4.1 Description of the subjects

During the period from 18.5.2020–2.8.2020, we recruited a total of 2,077 experimental subjects. The vast majority of our subjects are university students (N = 1,457). Among them, there are 208 healthcare students. We recruited 367 professionals from the non-healthcare sector. Our sample contains 213 healthcare professionals (of which 128 subjects associated themselves as a physician). The description of the subjects is depicted in Table 2 (for details on the variables and their measurement, see Appendix A2). In the following, we do not attempt to describe the variables in detail, but rather to communicate a broad sense of the data set. This is sufficient (but also necessary) to better understand the (regression) results which we describe later. To our surprise, a considerable number of people whom we approached with the request to advertise for the study attended themselves. Therefore, the level of education of the non-healthcare professionals is relatively high. This is important since non-healthcare professionals are a significant part of our control group in the population analysis. There are several differences between professionals and students. It is no surprise that professionals are on average older than students. However, there are interesting differences (in the willingness to attend the study) in gender. While slightly less than 50% of the professionals identified themselves as women, there was a surplus of women among the students. This surplus was particularly evident among the healthcare students. Moreover, we consider subjects who identified themselves with the third gender. However, its sample size is only low and, thus, any results are preliminary.

**Table 2:** Description of the subjects ^(1)^.

Variable		Total sample		Non-healthcare students		Healthcare students		Non-healthcare professionals		Healthcare professionals	
**Socio-demographic variables**											
Age (in years)		29.7979	12.3334	23.6909	4.6820	24.1730	4.1932	41.8770	11.8063	46.7934	13.3845
	Women	0.5840	-	0.5860	-	0.8413	-	0.4931	-	0.4741	-
Gender	Male	0.4028	-	0.3995	-	0.1442	-	0.4959	-	0.5211	-
	Diverse	0.0130	-	0.0144	-	0.0144	-	0.0108	-	0.0046	-
Education (0–5)		2.8395	1.1268	2.3394	0.5829	2.3605	0.7221	3.9209	1.2132	4.3286	0.9976
East (yes = 1)		0.3710	-	0.4243	-	0.2740	-	0.2861	-	0.3098	-
Household > 60 (yes = 1)		0.1377	-	0.1222	-	0.1497	-	0.1212	-	0.1800	-
Immunosuppression (yes = 1)		0.1074	-	0.0760	-	0.0817	-	0.1525	-	0.2112	-
Immunosuppression family (yes = 1)		0.5744	-	0.5924	-	0.5961	-	0.5476	-	0.5023	-
Smoker (yes = 1)		0.1190	-	0.1248	-	0.0769	-	0.1144	-	0.1267	-
**Anxiety and personal experiences**											
Quality health system (0, …, 10)		7.8375	1.5222	7.8494	1.4756	7.4471	1.6172	8.0000	1.4949	7.9061	1.6880
Worry virus (1, …, 7)		4.0337	1.8292	4.0440	1.8179	3.9711	1.8307	4.0544	1.8309	3.9530	1.8651
Worry isolation (1, …, 7)		4.2067	1.8632	4.2826	1.8668	4.3942	1.6992	3.8664	1.8409	4.1784	1.9561
Worry economy (1, …, 7)		4.9783	1.7345	5.0448	1.6581	4.9038	1.7167	4.7847	1.9119	5.0046	1.8313
Reaction food (1, …, 7)		2.7942	1.8135	2.6733	1.7492	2.4567	1.6471	3.2752	1.8898	2.9295	1.9904
Reaction disinfection (1, …, 7)		2.4987	1.6827	2.3554	1.6038	2.1730	1.4442	2.8855	1.8040	2.8638	1.8897
Reaction homeopathy (1, …, 7)		1.3175	0.9095	1.3170	0.8926	1.3076	0.8800	1.3378	0.9525	1.2957	0.9818
**Information**											
Mediaconsume today (0, …, 5)		1.2838	0.7599	1.2137	0.6921	1.1538	0.5941	1.4359	0.8905	1.5352	0.9188
Trustmedia (1, …, 5)		3.1190	0.9950	3.1224	0.9583	3.0048	1.0238	3.3133	1.0040	2.9436	1.1059
Trustmedia change (1, …, 5)		2.7990	0.6152	2.8158	0.5784	2.7307	0.6095	2.8801	0.5647	2.6572	0.8183
Statement overload (1, …, 7)		3.8096	1.6891	4.0104	1.6483	4.1971	1.5524	3.2561	1.6693	3.2018	1.7215
Statement fake news (1, …, 7)		4.9166	1.5886	4.9471	1.5695	5.1153	1.4927	4.6566	1.6552	5.0375	1.6450
Statement healthorganizations (1, …, 7)		4.9277	1.4208	4.9967	1.3640	5.0240	1.3019	4.8474	1.4742	4.6901	1.6732
Statement government (1, …, 7)		4.6428	1.4305	4.7021	1.3888	4.5913	1.3800	4.6757	1.4357	4.3896	1.6262
**Thinking/Reasoning/Effort**											
CRT (0, …, 3)		1.9797	1.0198	2.0016	1.0346	1.6875	1.0372	2.1416	0.9703	1.8826	0.9317
AOT (0, …, 7)		5.7982	0.7017	5.7451	0.7096	5.7506	0.6771	5.9529	0.6699	5.9336	0.6532
Duration participation (in minutes)		23.3321	11.1306	22.9144	10.1774	22.5051	10.9383	23.6221	12.6003	25.4684	13.1612

Means values left, Standard deviations on the right-hand side.

### 4.2 Analysis of the decision behavior

#### 4.2.1 First view on the decision behavior

Overall, the healthcare professionals performed slightly better than the students in distinguishing between true and false news stories (Table 3). Non-healthcare professionals performed best but the gap to the other populations is quite small. Within the stories, the performance of the subpopulations is similar to each other. Story three is outstanding in spite of the worse performance of all subpopulations. A first educated guess is that there is a gap between the viral narrative (that there are shortages of medical goods and commodities, such as toilet paper and disinfect, which has been reported by the media) and the key message of the story (reliance on foreign countries in spite of medical goods cannot be empirically proven).

**Table 3:** Fraction of correct identification–overall and story by story (N = 2,074) ^(1)^.

Variable	Total sample	Non-healthcare students	Healthcare students	Non-healthcare Professionals	Healthcare professionals
Overall (Stories 1–8)	64.54	63.55	63.34	68.15	65.31
Story 1: East Germany and Corona	55.18	54.28	50.00	61.85	53.05
Story 2: Psychiatrists on social isolation	89.92	88.79	92.78	90.73	92.95
Story 3: Germany’s medical care dependency	30.60	30.18	32.69	35.14	23.94
Story 4: Beds in clinics	51.08	49.95	47.59	53.40	55.86
Story 5: Gender and Corona	70.65	68.61	66.82	79.01	69.95
Story 6: Efficacy of homeopathy	88.62	87.59	88.46	91.28	91.07
Story 7: Corona App	59.90	59.16	61.05	59.12	63.38
Story 8: Corona transmission by farts	70.40	69.81	67.30	74.65	72.30

^1)^ The values are presented in percent.

We want to mention another point that is related to the performance of healthcare professionals in the stories 5 and 6, i.e., the stories that address direct health implications due to Covid-19 and in which we could have expected the medical professionals to perform much better than the other ones. However, the healthcare professionals performed quite similar to the other subpopulations (or only slightly better). One reason for this might be that they “speak another language.” In other words, they might have perceived everyday articles from the media as incorrect due to the chosen words of the author of the news story.

#### 4.2.2 Regression analysis

*I*. *Overall correct identification*. The regression results are depicted in Table 4. Panel Ia is our main specification, which we want to describe in detail. The other two estimations serve as a robustness check. *Healthcare professionals* (β = -0.2635, p-value = 0.019) *seem* to perform less well than students with (β = 0.0339, p-value = 0.800) and without (β = 0.0127, p-value = 0.906) healthcare background. Its sign is negative, whereas the student variables are slightly but positively associated with the overall correct identification of corona-related news stories. *CRT* and *AOT* are positively related with the ability to correctly distinguish between true and false. The magnitude of *CRT* (β = 0.0886, p-value = 0.003) seems to be more pronounced than *AOT* (β = 0.0686, p-value = 0.124). The association of *Familiarity* with the stories and correct identification is positive, but very small (β = 0.0177, p-value = 0.514). The same pattern can be observed for the variable *Surprising* (β = 0.0136, p-value = 0.425). A somewhat larger effect results from *Certainty* (β = 0.0381, p-value = 0.040). Overall, subjects from *East* Germany do not seem to differ much from the other subjects. Its sign is positive, but the effect size is quite small in magnitude (β = 0.0467, p-value = 0.461). The associations between the variables *Quality health system* to *Smoker* of the regression output (which capture the subjects’ anxiety and personal experiences) and the ability to distinguish between true and false show no clear pattern. They all have in common that the effect size is relatively low, but the sign of the respective variables varies seemingly at random. Interestingly, *Age* is positively related to our variable of interest (β = 0.0405, p-value = 0.019). However, if people get older the effect declines (β = -0.0449, p-value = 0.027). The other variables are addressed exploratory. We only refer to these variables if they seem to have a large contribution to the distinction between true and false. Our focus is on education and time spent to finish the study. *Education* seems to have a considerable explanatory power for our research interest. The coefficient is positive and its magnitude is large (β = 0.1042, p-value = 0.005). The more time the subjects took to finish the study (i.e., *Duration participation*) the better they were in distinguishing between true and false (β = 0.0217, p-value = 0.012). But as subjects took more time the effect diminishes (β = -0.0218, p-value = 0.072).

**Table 4:** OLS regressions to explain “Overall correct identification” (N = 2,053).

Y = 0–8, overall correctly identified stories			Ia		IIa		IIIa	
			dy/dx (Std. Err.)	P>|t|	dy/dx (Std. Err.)	P>|t|	dy/dx (Std. Err.)	P>|t|
Population		Healthcare professionals	-0.2635 (0.1119)	0.019	-0.2145 (0.1107)	0.053	-0.2172 (0.1099)	0.048
		Healthcare students	0.0339 (0.1340)	0.800	-0.0432 (0.1315)	0.742	-0.2296 (0.1140)	0.044
		Non-healthcare students	0.0127 (0.1078)	0.906	-0.0677 (0.1042)	0.516	-0.2720 (0.0762)	< 0.001
CRT			0.0886 (0.0299)	0.003	0.0937 (0.0299)	0.002	0.0884 (0.0299)	0.003
AOT			0.0686 (0.0445)	0.124	0.0837 (0.0443)	0.059	0.0854 (0.0444)	0.055
Certainty			0.0381 (0.0185)	0.040	0.0382 (0.0186)	0.040	0.0392 (0.0186)	0.035
Familiarity			0.0177 (0.0271)	0.514	0.0164 (0.0272)	0.546	0.0118 (0.0271)	0.665
Surprising			0.0136 (0.0171)	0.425	0.0135 (0.0171)	0.430	0.0050 (0.0170)	0.767
East (yes = 1)			0.0467 (0.0632)	0.461	0.0467 (0.0633)	0.461	0.0435 (0.0634)	0.493
Quality health system			0.0177 (0.0193)	0.361	0.0219 (0.0193)	0.256	0.0185 (0.0193)	0.337
Worry virus			-0.0049 (0.0169)	0.770	-0.0037 (0.0169)	0.826	-0.0021 (0.0170)	0.901
Worry isolation			0.0131 (0.0159)	0.411	0.0146 (0.0159)	0.359	0.0133 (0.0159)	0.405
Worry economy			-0.0251 (0.0168)	0.134	-0.0233 (0.0168)	0.166	-0.0232 (0.0168)	0.168
Reaction food			0.0191 (0.0213)	0.371	0.0196 (0.0214)	0.359	0.0260 (0.0213)	0.222
Reaction disinfection			-0.0196 (0.0238)	0.411	-0.0187 (0.0239)	0.433	-0.0214 (0.0239)	0.370
Reaction homeopathy			-0.0042 (0.0332)	0.899	-0.0036 (0.0333)	0.915	0.0040 (0.0332)	0.905
Household (>60)			-0.0915 (0.0856)	0.285	-0.0911 (0.0858)	0.288	-0.1177 (0.0820)	0.151
Immunosuppression			0.0821 (0.0934)	0.380	0.0676 (0.0935)	0.469	0.0883 (0.0926)	0.341
Immunosuppression family			-0.0305 (0.0576)	0.596	-0.0380 (0.0576)	0.510	-0.0345 (0.0577)	0.550
Smoker (yes = 1)			0.0304 (0.0871)	0.727	0.0106 (0.0870)	0.903	0.0270 (0.0870)	0.756
Age			0.0405 (0.0173)	0.019	0.0533 (0.0167)	0.001	-(-)	-
Age squared			-0.0449 (0.0203)	0.027	-0.0571 (0.0198)	0.004	-(-)	-
Education			0.1042 (0.0369)	0.005	-(-)	-	-(-)	-
Gender	Male		-0.0519 (0.0629)	0.410	-0.0456 (0.0630)	0.470	-0.0384 (0.0629)	0.542
	Diverse		-0.2060 (0.2445)	0.400	-0.2058 (0.2449)	0.401	-0.1848 (0.2454)	0.452
Mediaconsume today			0.0218 (0.0381)	0.566	0.0145 (0.0380)	0.702	0.0204 (0.0380)	0.592
Trustmedia			0.0370 (0.0350)	0.291	0.0426 (0.0350)	0.224	0.0453 (0.0350)	0.196
Trustmedia change			0.0304 (0.0546)	0.578	0.0276 (0.0547)	0.614	0.0365 (0.0548)	0.505
Statement overload			-0.0374 (0.0187)	0.045	-0.0387 (0.0187)	0.038	-0.0414 (0.0187)	0.027
Statement fake news			0.0028 (0.0188)	0.881	0.0021 (0.0189)	0.912	0.0020 (0.0189)	0.915
Statement healthorganizations			-0.0421 (0.0359)	0.241	-0.0444 (0.0359)	0.217	-0.0461 (0.0360)	0.201
Statement government			0.0288 (0.0357)	0.420	0.0283 (0.0357)	0.429	0.0290 (0.0358)	0.418
Duration participation (minutes)			0.0217 (0.0086)	0.012	0.0202 (0.0086)	0.019	0.0219 (0.0086)	0.011
Duration squared			-0.0218 (0.0121)	0.072	-0.0204 (0.0121)	0.092	-0.0221 (0.0121)	0.068
Week			-0.0089 (0.0130)	0.492	-0.0087 (0.0130)	0.506	-0.0078 (0.0130)	0.548
Prob > F (Adj R-squared)			0.0000 (0.0355)		0.0000 (0.0322)		0.0000 (0.0276)	

A robustness check revealed interesting insights: If we refrain from controlling for *Education* and *Age*, there are changes in the variables *Population* and *AOT* (the other variables remain the same). Most notably, the seemingly worse performance of the *Healthcare professionals* (compared to students with and without healthcare background) vanishes. In contrast, they even perform slightly better. Moreover, the magnitude of *AOT* increases. Considering panel IIa, the drivers of the effects seem to be mainly *Education* in the case of *AOT*, and *Age* in the case of the *Healthcare professionals* and *students*.

In the following story-by-story analysis, we check our regressions if there is a change if we do not control for age or/and education. If so, then we will report it. Otherwise, we stick to our main regression.

*II*. *Story-by-story correct identification*. In this section, we want to present insights that are related to the individual stories. We focus on *some highlights* for each story only (Tables 5 and 6).

**Table 5:** Marginal effects after logit regressions to explain “Correct identification” (N = 2,053).

Y = 1, correctly identified Y = 0, else			Ib		IIb		IIIb		IVb	
			dy/dx (Std. Err.)	P>|z|	dy/dx (Std. Err.)	P>|z|	dy/dx (Std. Err.)	P>|z|	dy/dx (Std. Err.)	P>|z|
Population		Healthcare professionals	-0.0953 (0.0440)	0.030	0.0111 (0.0308)	0.718	-0.0958 (0.0379)	0.011	-0.0096 (0.0433)	0.824
		Healthcare students	-0.0148 (0.0523)	0.777	0.0548 (0.0306)	0.074	-0.0030 (0.0490)	0.951	0.0533 (0.0513)	0.299
		Non-healthcare students	0.0026 (0.0422)	0.951	0.0236 (0.0277)	0.395	-0.0357 (0.0389)	0.359	0.0618 (0.0411)	0.133
CRT			0.0170 (0.0116)	0.143	0.0129 (0.0067)	0.053	-0.0187 (0.0105)	0.076	0.0108 (0.0116)	0.351
AOT			-0.0066 (0.0174)	0.703	0.0136 (0.0096)	0.157	-0.0274 (0.0157)	0.080	0.0102 (0.0173)	0.556
Certainty			0.0044 (0.0043)	0.300	0.0256 (0.0025)	< 0.001	-0.0331 (0.0039)	< 0.001	0.0054 (0.0043)	0.211
Familiarity			0.1875 (0.0515)	< 0.001	0.0361 (0.0232)	0.119	-0.1841 (0.0349)	< 0.001	0.2678 (0.0399)	< 0.001
Surprising			0.0393 (0.0220)	0.074	0.0160 (0.0137)	0.245	-0.0330 (0.0226)	0.145	0.0132 (0.0228)	0.564
East (yes = 1)			0.0699 (0.0247)	0.005	-0.0100 (0.0141)	0.478	0.0081 (0.0222)	0.716	0.0182 (0.0246)	0.458
Quality health system			-0.0035 (0.0076)	0.642	0.0050 (0.0042)	0.233	0.0168 (0.0070)	0.016	0.0078 (0.0076)	0.305
Worry virus			0.0005 (0.0066)	0.945	0.0034 (0.0039)	0.379	-0.0020 (0.0060)	0.739	-0.0123 (0.0066)	0.061
Worry isolation			-0.0123 (0.0062)	0.048	0.0091 (0.0036)	0.011	-0.0024 (0.0056)	0.672	0.0185 (0.0062)	0.003
Worry economy			0.0043 (0.0066)	0.511	-0.0042 (0.0038)	0.272	0.0000 (0.0059)	0.997	-0.0097 (0.0065)	0.136
Reaction food			0.0092 (0.0083)	0.270	0.0037 (0.0050)	0.461	0.0035 (0.0075)	0.644	0.0002 (0.0083)	0.979
Reaction disinfection			-0.0089 (0.0093)	0.339	-0.0114 (0.0055)	0.037	0.0011 (0.0085)	0.894	-0.0080 (0.0093)	0.392
Reaction homeopathy			0.0122 (0.0131)	0.349	0.0027 (0.0073)	0.710	0.0100 (0.0118)	0.397	-0.0176 (0.0131)	0.179
Household (> 60)			-0.0399 (0.0333)	0.231	-0.0173 (0.0182)	0.340	-0.0736 (0.0321)	0.022	0.0194 (0.0333)	0.561
Immunosuppression			0.0438 (0.0369)	0.236	-0.0020 (0.0222)	0.929	-0.0085 (0.0340)	0.804	0.0750 (0.0369)	0.042
Immunosuppression family			0.0037 (0.0225)	0.869	0.0180 (0.0130)	0.167	-0.0171 (0.0202)	0.397	-0.0099 (0.0224)	0.660
Smoker (yes = 1)			0.0358 (0.0344)	0.298	0.0382 (0.0217)	0.079	-0.0137 (0.0309)	0.658	-0.0322 (0.0339)	0.343
Age			0.0117 (0.0068)	0.083	0.0041 (0.0041)	0.317	-0.0033 (0.0063)	0.603	0.0140 (0.0068)	0.038
Age squared			-0.0123 (0.0079)	0.121	-0.0028 (0.0049)	0.570	0.0022 (0.0075)	0.768	-0.0163 (0.0080)	0.041
Education			0.0108 (0.0145)	0.458	-0.0050 (0.0093)	0.586	0.0234 (0.0134)	0.080	0.0311 (0.0144)	0.031
Gender	Male		-0.0197 (0.0246)	0.424	-0.0355 (0.0148)	0.016	0.0728 (0.0224)	0.001	-0.0026 (0.0245)	0.914
	Diverse		0.0226 (0.0943)	0.810	-0.0173 (0.0628)	0.783	0.0279 (0.0864)	0.747	0.0800 (0.0927)	0.388
Mediaconsume today			0.0117 (0.0149)	0.433	0.0193 (0.0097)	0.047	0.0020 (0.0135)	0.882	-0.0007 (0.0149)	0.963
Trustmedia			0.0011 (0.0136)	0.937	-0.0013 (0.0079)	0.872	-0.0071 (0.0124)	0.565	0.0064 (0.0136)	0.638
Trustmedia change			0.0280 (0.0213)	0.190	0.0165 (0.0124)	0.185	-0.0039 (0.0196)	0.842	-0.0352 (0.0215)	0.101
Statement overload			-0.0076 (0.0073)	0.292	-0.0071 (0.0044)	0.108	-0.0026 (0.0066)	0.691	-0.0158 (0.0072)	0.029
Statement fake news			-0.0065 (0.0074)	0.376	0.0019 (0.0044)	0.664	-0.0034 (0.0067)	0.614	0.0037 (0.0074)	0.618
Statement healthorganizations			-0.0101 (0.0141)	0.472	-0.0072 (0.0080)	0.374	0.0004 (0.0128)	0.975	-0.0238 (0.0140)	0.089
Statement government			0.0140 (0.0140)	0.317	0.0040 (0.0080)	0.618	0.0010 (0.0128)	0.940	0.0112 (0.0139)	0.418
Duration participation (minutes)			0.0017 (0.0035)	0.618	-0.0012 (0.0018)	0.508	0.0092 (0.0030)	0.002	-0.0011 (0.0034)	0.752
Duration squared			-0.0001 (0.0050)	0.978	-0.0004 (0.0024)	0.863	-0.0097 (0.0043)	0.023	0.0027 (0.0048)	0.578
Week			-0.0033 (0.0051)	0.524	-0.0040 (0.0030)	0.181	-0.0030 (0.0046)	0.515	0.0082 (0.0051)	0.102
Prob > chi2 (Pseudo R2)			0.0000 (0.0283)		0.0000 (0.1448)		0.0000 (0.0764)		0.0000 (0.0414)	

**Table 6:** Marginal effects after logit regressions to explain “Correct identification” (N = 2,053), cont.

Y = 1, correctly identified Y = 0, else			Vb		VIb		VIIb		VIIIb	
			dy/dx (Std. Err.)	P>|z|	dy/dx (Std. Err.)	P>|z|	dy/dx (Std. Err.)	P>|z|	dy/dx (Std. Err.)	P>|z|
Population		Healthcare professionals	-0.1082 (0.0416)	0.009	-0.0281 (0.0334)	0.400	0.0553 (0.0429)	0.197	-0.0045 (0.0414)	0.914
		Healthcare students	-0.0920 (0.0461)	0.046	0.0082 (0.0321)	0.798	0.0334 (0.0520)	0.521	-0.0154 (0.0487)	0.752
		Non-healthcare students	-0.0651 (0.0356)	0.067	0.0037 (0.0266)	0.889	0.0135 (0.0422)	0.748	0.0072 (0.0390)	0.854
CRT			0.0008 (0.0103)	0.939	0.0163 (0.0068)	0.015	0.0109 (0.0115)	0.345	0.0392 (0.0103)	< 0.001
AOT			0.0181 (0.0155)	0.243	0.0122 (0.0100)	0.222	-0.0175 (0.0173)	0.311	0.0418 (0.0155)	0.007
Certainty			0.0304 (0.0032)	< 0.001	0.0227 (0.0022)	< 0.001	0.0171 (0.0041)	< 0.001	0.0168 (0.0032)	< 0.001
Familiarity			0.2337 (0.0438)	< 0.001	0.0176 (0.0361)	0.626	-0.0574 (0.0411)	0.162	-0.0819 (0.0500)	0.101
Surprising			0.0148 (0.0202)	0.464	-0.0322 (0.0140)	0.021	0.0464 (0.0270)	0.086	-0.0372 (0.0234)	0.111
East (yes = 1)			-0.0101 (0.0220)	0.645	-0.0202 (0.0148)	0.172	0.0103 (0.0245)	0.674	-0.0088 (0.0225)	0.695
Quality health system			-0.0131 (0.0068)	0.053	0.0033 (0.0044)	0.449	-0.0019 (0.0075)	0.798	-0.0013 (0.0069)	0.846
Worry virus			0.0016 (0.0059)	0.783	0.0063 (0.0041)	0.126	0.0063 (0.0066)	0.334	-0.0080 (0.0061)	0.189
Worry isolation			0.0008 (0.0056)	0.892	0.0025 (0.0038)	0.511	-0.0015 (0.0062)	0.813	-0.0035 (0.0057)	0.539
Worry economy			-0.0073 (0.0059)	0.217	0.0019 (0.0040)	0.638	-0.0043 (0.0065)	0.512	-0.0043 (0.0060)	0.479
Reaction food			0.0092 (0.0076)	0.221	0.0081 (0.0053)	0.131	-0.0091 (0.0083)	0.271	-0.0063 (0.0077)	0.408
Reaction disinfection			-0.0005 (0.0085)	0.954	-0.0138 (0.0057)	0.015	0.0119 (0.0093)	0.201	0.0131 (0.0086)	0.128
Reaction homeopathy			-0.0049 (0.0118)	0.675	-0.0155 (0.0064)	0.016	0.0040 (0.0131)	0.76	0.0132 (0.0119)	0.265
Household (> 60)			-0.0370 (0.0298)	0.214	0.0152 (0.0206)	0.460	0.0716 (0.0340)	0.035	-0.0317 (0.0298)	0.287
Immunosuppression			-0.0247 (0.0330)	0.454	-0.0247 (0.0214)	0.249	-0.0232 (0.0363)	0.522	0.0507 (0.0345)	0.141
Immunosuppression family			-0.0204 (0.0201)	0.310	-0.0185 (0.0139)	0.184	-0.0287 (0.0223)	0.198	0.0324 (0.0205)	0.114
Smoker (yes = 1)			-0.0547 (0.0295)	0.064	0.0139 (0.0209)	0.507	-0.0048 (0.0336)	0.887	0.0593 (0.0319)	0.063
Age			0.0040 (0.0061)	0.515	-0.0030 (0.0042)	0.482	0.0060 (0.0067)	0.371	0.0028 (0.0061)	0.640
Age squared			-0.0025 (0.0072)	0.733	0.0034 (0.0051)	0.512	-0.0067 (0.0079)	0.399	-0.0054 (0.0071)	0.445
Education			-0.0167 (0.0136)	0.222	0.0271 (0.0085)	0.001	-0.0047 (0.0145)	0.743	0.0271 (0.0132)	0.040
Gender	Male		-0.0159 (0.0221)	0.472	-0.0114 (0.0158)	0.471	-0.0418 (0.0244)	0.087	-0.0057 (0.0227)	0.803
	Diverse		-0.2155 (0.0918)	0.019	0.0573 (0.0489)	0.241	-0.1940 (0.0949)	0.041	-0.0611 (0.0918)	0.506
Mediaconsume today			0.0035 (0.0135)	0.793	0.0036 (0.0091)	0.691	-0.0092 (0.0147)	0.532	-0.0064 (0.0134)	0.631
Trustmedia			0.0044 (0.0121)	0.715	0.0046 (0.0084)	0.587	0.0305 (0.0135)	0.024	-0.0062 (0.0126)	0.625
Trustmedia change			0.0334 (0.0189)	0.077	0.0017 (0.0128)	0.895	-0.0225 (0.0212)	0.288	0.0267 (0.0193)	0.167
Statement overload			0.0027 (0.0065)	0.677	0.0003 (0.0046)	0.955	0.0010 (0.0072)	0.893	-0.0021 (0.0067)	0.757
Statement fake news			0.0053 (0.0066)	0.424	0.0091 (0.0046)	0.047	-0.0038 (0.0073)	0.602	-0.0008 (0.0068)	0.903
Statement healthorganizations			-0.0123 (0.0126)	0.329	0.0018 (0.0087)	0.839	-0.0012 (0.0139)	0.934	0.0123 (0.0128)	0.336
Statement government			0.0047 (0.0125)	0.706	-0.0037 (0.0087)	0.673	-0.0009 (0.0138)	0.946	-0.0004 (0.0128)	0.973
Duration participation (minutes)			0.0030 (0.0031)	0.326	0.0014 (0.0021)	0.497	0.0048 (0.0034)	0.155	0.0054 (0.0030)	0.075
Duration squared			-0.0035 (0.0043)	0.417	-0.0011 (0.0029)	0.694	-0.0045 (0.0048)	0.345	-0.0074 (0.0042)	0.076
Week			-0.0022 (0.0045)	0.621	-0.0022 (0.0031)	0.473	0.0024 (0.0051)	0.629	-0.0040 (0.0047)	0.390
Prob > chi2 (Pseudo R2)			0.0000 (0.0733)		0.0000 (0.1395)		0.0000 (0.0186)		0.0000 (0.0385)	

*Story 1*. The key driver to explain the ability to distinguish between true and false news is *Familiarity* (β = 0.1875, p-value<0.001). The *East*-Dummy is positive, i.e., subjects from East Germany performed better in this story (β = 0.0699, p-value = 0.005). This is in line with a confirmation bias: people from each of both regions might have thought that they are better prepared for the corona crisis. But only in the case of East-Germany, this answering behavior is associated with a correct answer (because Haseloff has actually said that the East is better prepared).

*Story 2*. *Male* perform slightly worse than women in distinguishing between true and false (β = -0.0355, p-value = 0.016). Possible explanations might include own experiences, empathy, and, in case of the students, training in their respective degree programs. Moreover, is interesting that *Certainty* increases the probability to perform well (β = 0.0256, p-value<0.001).

*Story 3*. In this story, *Familiarity* (β = -0.1841, p-value<0.001) and (to a less extent) *Certainty* (β = -0.0331, p-value<0.001) are drivers of the decision behavior of the subjects. Both signs are negative and the magnitude of the variables seems to be important. Probably the subjects have heard about news stories in this realm. We guess that the pervasive narrative (i.e., there is a shortage of medical goods and commodities as well as a considerable reliance on foreign countries) contradicts the finding of the correct version of the news story. This is in line with a confirmation bias.

*Story 4*. *Familiarity* is a strong predictor in this story (β = 0.2678, p-value<0.001). If people believe that they know the story they do perform better. *Immunosuppression* is positively related to the variable of interest (β = 0.0750, p-value = 0.042). The effect size is large. This is interesting because one might have expected that this group of people fears a lack of capacities of hospitals most. Maybe the unexpected finding can be explained by the experience of regular visits to physicians and hospitals.

*Story 5*. In this story, *Familiarity* is a strong predictor in this story (β = 0.2337, p-value<0.001). Compared to women, subjects who identified themselves as *Diverse* performed less well in distinguishing between true and false (β = -0.2155, p-value = 0.019). This result is at best preliminary since the sample size is small.

*Story 6*. As expected, *Reaction homeopathy* is negatively (but only slightly) associated with our variable of interest (β = -0.0155, p-value = 0.016). This is in line with a confirmation bias: The correction version of this story was that homeopathic remedies themselves have no effect. People who have stronger used homeopathic stuff probably did not exclude that there is a positive effect. The effect of *Reaction disinfection* (β = -0.0138, p-value = 0.015) is similar to *Reaction homeopathy*.

*Story 7*. Small, positive effects can be observed in the variables *Surprising* (β = 0.0464, p-value = 0.086) and (to a less extent) *Certainty* (β = 0.0171, p-value<0.001). The variable *Trustmedia* (β = 0.0305, p-value = 0.024) has a considerable positive effect. Compared to women, subjects who identified themselves as *Diverse* performed less well in distinguishing between true and false (β = -0.1940, p-value = 0.041).

*Story 8*. *CRT* (β = 0.0392, p-value<0.001) and *AOT* (β = 0.0418, p-value = 0.007) prevent of falling for this kind of strong exaggeration.

## 5 Conclusions and discussion

Infodemics–the spread of false news claims–constitutes a great societal challenge during the corona crisis. This paper addressed the question of who is good at distinguishing between true and false news stories in the realm of corona. For this purpose, we recruited not only students but also healthcare professionals. The main findings of the study can be summarized as follows: We find that healthcare professionals, non-healthcare professionals, and students both with and without healthcare background perform similarly in distinguishing between true and false news stories. Moreover, we find that the residence of the subjects (East- or West-Germany) plays only a minor role. Furthermore, we found evidence that the propensity to engage in analytical thinking (CRT) and actively open-minded thinking (AOT) are positively associated with the ability to correctly distinguish between true and false news stories. When this study was carried out, there was a shortage of commodities (most notably, the pictures where toilet paper and disinfect was out of sale went viral in the German media). Probably as a consequence of having this picture in mind, people incorrectly thought that Germany’s medical care heavily depends on foreign countries. If news stories are in line with existing narratives, subjects tend to think that the stories are true.

With regard to CRT and AOT, our results are in line with Pennycook and Rand [14,15]. They also found that these two determinants help to distinguish between true and false news stories. Our finding that narratives seem to matter is related to the literature of the confirmation bias [24]. Confirmation bias is about prior beliefs that influence if individuals agree to something or not. Narratives are stories that go viral at a specific time. They may influence the beliefs of individuals. To the best of our knowledge, the other two findings have not been systematically studied before. Overall, the residence of the subjects (East- or West-Germany) does not seem to matter much for our topic. Maybe about 30 years after reunification, the different socialization is not an important point when it comes to distinguishing between true and false news stories. To our surprise, healthcare professionals did not perform better than non-healthcare professionals or students even if the news stories were linked to immediate health implications. Maybe healthcare professionals speak in another language than other people, and perceive everyday articles from the media as incorrect due to the wording of the author who tries to reach a broad audience.

Our study shows that individuals are vulnerable to false news information, regardless of their level of education and expertise. In this realm, narratives seem to matter: communication of the mass media influences people’s perception of the state of the world. We identified AOT and CRT as protective factors: teaching activities in this area might help to better distinguish between true and false news stories and, in turn, help to reduce the spread of false news stories. However, our study suffers from some limitations. For example, we deal with a non-representative convenience sample with mostly high-educated individuals–further research should address the general population. Furthermore, the role of narratives should further be investigated. For example, it is important to find out how narratives and the perception of news stories (either true or false) are correlated with each other. Moreover, it would add value to the literature to find out under which circumstances people think about news stories, accept them uncritically or even ignore it.

## Appendix

**Table A1:** Source of the news stories of the experiment.

**1**	https://www.sueddeutsche.de/gesundheit/krankheiten-magdeburg-haseloff-ostdeutsche-besser-auf-corona-krise-vorbereitet-dpa.urn-newsml-dpa-com-20090101-200331-99-543961
**2**	https://www.berliner-kurier.de/panorama/psychiater-warnen-vor-ansteigender-suizidrate-bei-laengerer-kontaktsperre-li.79446
**3**	https://www.faz.net/aktuell/wirtschaft/deutsche-medizinversorgung-ist-gar-nicht-so-abhaengig-von-asien-16727733.html
**4**	https://www.tagesschau.de/investigativ/ndr/krankenhaeuser-kurzarbeit-101.html
**5**	https://www.bild.de/ratgeber/gesundheit/gesundheit/studie-aus-niederlanden-ein-enzym-macht-maenner-anfaelliger-fuer-corona-70577794.bild.html
**6**	https://www.medizin-transparent.at/coronavirus-globuli
**7**	https://www.tagesschau.de/investigativ/swr/tracking-app-101.html
**8**	https://www.swr3.de/aktuell/nachrichten/Australischer-Arzt-warnt-vor-Corona-Uebertragung-durch-Fuerze/-/id=47428/did=5606260/18xdh0a/index.html

**Table A2:** Codebook of the collected variables and their measurement.

**Variable**	**Question/Statement**	**Values**
Age	How old are you?	18–99
AOT	7-item scale from Haran et al. (2013:201)	1 = completely disagree, 7 = completely agree (last 4 items reversed coded)
Certainty	How certain are you?	0 = guessed, …, 10 = entirely certain
Correctly identified	Is this news story true or false?	1 = correctly identified (i.e., true story identified as true and false story identified as false), 0 = else
CRT 1	A safety mask and a disinfectant product cost together €11.10. The protective mask costs €10 more than the disinfectant. How much does the disinfectant cost?	1 (correct answer) if = €0.55, 0 else
CRT 2	Five machines need five minutes to produce five protective masks. How long do 100 machines need to produce 100 protective masks?	1 (correct answer) if 5, 0 else
CRT 3	A virus is spreading in a city. Every day the number of infected people doubles. It takes 48 days for the virus to infect the entire population. How long would it take for the virus to infect half of the population?	1 (correct answer) if 47, 0 else
Duration	-	Time spent to complete the survey (in minutes)
East	In which federal state do you live?	East = 1 if Saxony or Saxony-Anhalt or Mecklenburg-Western Pomerania or Thuringia or Brandenburg, West = 0 if else
Education	Please, indicate your highest level of education.	Increasing from low education (e.g. no degree) to high degree (PhD) on a scale from 0 to 5
Familiarity	Have you seen or heard of this news story before?	1 = yes, 0 = else
Gender	What gender would you associate yourself with?	0 = Female, 1 = Male, 2 = Diverse
Household > 60	Do people over 60 live in your household?	1 = yes, 0 = else
Immunosuppression	Is your immune system weakened (e.g. by the intake of certain drugs) and/or do you suffer from at least one of the following pre-existing conditions: Disease of the cardiovascular system (e.g. coronary heart disease and high blood pressure), diseases of the lungs (e.g. COPD), chronic liver disease, diabetes mellitus (diabetes), cancer	1 = yes, 0 = else
Immunosuppression family	Is the immune system of a person from your close family and/or circle of friends weakened or does such a person suffer from one of the above-mentioned pre-existing conditions?	1 = yes, 0 = else
Mediaconsume today	In the last 7 days, how much time have you spent on average per day consuming information related to COVID-19 (e.g. watching/reading news, researching case numbers, etc.)	0 = No time, 1 = Less than 1 hour, 2 = 1 hour to under 2 hours, 3 = 2 hours to less than 3 hours, 4 = 3 hours to less than 4 hours, 5 = More than 4 hours
Population	I am currently. . .	1 = working in the healthcare sector. 2 = working in the non-healthcare sector. 3 = enrolled as a student in a 4 = college/university. 5 = not employed. 6 = retired. 7 = other Follow up question: If identified as a student: healthcare sector or not
Quality health system	How do you evaluate the quality of the German healthcare system in general?	0 = very worse, …, 10 = very good
Reaction food	Indicate the extent to which you agree with the following statements: In response to COVID-19 (coronavirus), I have built up a reserve of staple foods.	1 = Not applicable at all, …, 7 = Fully applicable
Reaction disinfection	Indicate the extent to which you agree with the following statements: In response to COVID-19 (coronavirus), I have created a reserve of hygiene articles and disinfection.	1 = Not applicable at all, …, 7 = Fully applicable
Reaction homeopathy	Indicate the extent to which you agree with the following statements: In response to COVID-19 (coronavirus) I have made increasing use of homeopathy and naturopathy.	1 = Not applicable at all, …, 7 = Fully applicable
Smoker	Do you smoke?	1 = yes, 0 = else
Statement fake news	Indicate how you evaluate the following statements in connection with COVID-19 (coronavirus): I’m worried about fake news.	1 = Not applicable at all, …, 7 = Fully applicable
Statement government	Indicate how you evaluate the following statements in connection with COVID-19 (coronavirus): I feel safe with the information I receive from the government.	1 = Not applicable at all, …, 7 = Fully applicable
Statement healthorganizations	Indicate how you evaluate the following statements in connection with COVID-19 (coronavirus): The information from health authorities and health institutes give me security.	1 = Not applicable at all, …, 7 = Fully applicable
Statement overload	Indicate how you evaluate the following statements in connection with COVID-19 (coronavirus): I feel overwhelmed by the amount of information.	1 = Not applicable at all, …, 7 = Fully applicable
Surprising	Did the news story surprise you while reading it?	1 = yes, 0 = else
Trustmedia	How much do you trust the mass media—such as newspapers, television and radio—to report the news completely, accurately and fairly?	1 = very low, …, 5 = very high
Trustmedia change	How has your trust in the mass media changed during the corona crisis?	1 = strongly decreased, …, 5 = strongly increased
Week	-	Participation in the first 7 days of the study = Week 1, Participation in the next 7 days of the study = Week 2, etc.
Worry economy	What are your concerns about COVID-19 (coronavirus)? Economic consequences.	1 = Not applicable at all, …, 7 = Fully applicable
Worry isolation	What are your concerns about COVID-19 (coronavirus)? Consequences for health due to the isolation or restriction of social contacts.	1 = Not applicable at all, …, 7 = Fully applicable
Worry virus	What are your concerns about COVID-19 (coronavirus)? Immediate health threat due to the virus.	1 = Not applicable at all, …, 7 = Fully applicable
